# CHARACTERISTICS OF BURDEN ASSOCIATED WITH CARING FOR OLDER FAMILY MEMBERS WITH DYSPHAGIA ACROSS TIME

**DOI:** 10.1093/geroni/igae098.0636

**Published:** 2024-12-31

**Authors:** Samantha Shune, Merle Werbeloff, Ashwini Namasivayam-MacDonald

**Affiliations:** University of Oregon, Eugene, Oregon, United States; University of the Witwatersrand, Johannesburg, Gauteng, South Africa; McMaster University, Hamilton, Ontario, Canada

## Abstract

Caregivers often experience increased burden resulting in reduced health and quality of life. Underexplored in its impact on burden is the effect of dysphagia (swallowing difficulties) over time. The purpose of this study was to explore the characteristics of burden associated with caring for an individual with dysphagia across an aging cohort. Using the Round 1, 5, 7, and 11 surveys from the National Health and Aging Trends Study and the National Study of Caregiving, we analyzed data from 4,662 spousal and children caregivers caring for community-dwelling family aged 65+. Outcome variables included caregivers’ emotional, financial, and physical burden and their agreement with statements about their well-being. A design-based conversion of the Pearson chi square statistic and binary logistic regressions were used to explain and predict presence of burden, its extent, and characteristics dependent on presence of dysphagia. At any given time, 16-27% of respondents were caring for family with dysphagia. Caregiver burden was more often reported by those caring for family with dysphagia than those without. For spouses and daughters, the degree of burden was almost always rated higher among those caring for family with dysphagia, with significant differences observed in levels of emotional and physical burden. Further, caregivers of family with dysphagia were significantly more likely to report having little time for themselves and feeling depressed, lonely, and that caregiving was too much to handle. Ultimately, dysphagia’s potential impact on the emotional, financial, and physical well-being of family caregivers must be considered along the care trajectory.

